# Composition of Strawberry Floral Volatiles and their Effects on Behavior of Strawberry Blossom Weevil, *Anthonomus rubi*

**DOI:** 10.1007/s10886-020-01221-2

**Published:** 2020-10-08

**Authors:** Raimondas Mozūraitis, David Hall, Nina Trandem, Baiba Ralle, Kalle Tunström, Lene Sigsgaard, Catherine Baroffio, Michelle Fountain, Jerry Cross, Atle Wibe, Anna-Karin Borg-Karlson

**Affiliations:** 1 Department of Zoology, Stockholm University, Stockholm, Sweden; 2 Natural Resources Institute, University of Greenwich, Chatham Maritime, Kent, ME4 4TB UK; 3 NIBIO, Norwegian Institute of Bioeconomy Research, NO-1431 Ås, Norway; 4 Latvian Plant Protection Research Centre, Riga, LV-1039 Latvia; 5 Department of Plant and Environmental Sciences, University of Copenhagen, 1871 Frederiksberg C, Denmark; 6 Agroscope, Research Center Conthey, 1964 Conthey, Switzerland; 7 NIAB EMR, East Malling, Kent, ME19 6BJ UK; 8 Norwegian Centre for Organic Agriculture, NO-6630 Tingvoll, Norway; 9 Department of Chemistry, School of Engineering Science in Chemistry, Biotechnology and Health, KTH, Royal Institute of Technology, 10044 Stockholm, Sweden; 10 Department of Chemical Engineering, Mid Sweden University, 85170 Sundsvall, Sweden

**Keywords:** *Anthonomus rubi*, *Fragaria x ananassa*, ***Fragaria vesca***, Floral odors, Semiochemicals, Pest control

## Abstract

The strawberry blossom weevil (SBW), *Anthonomus rubi*, is a major pest in strawberry fields throughout Europe. Traps baited with aggregation pheromone are used for pest monitoring. However, a more effective lure is needed. For a number of pests, it has been shown that the attractiveness of a pheromone can be enhanced by host plant volatiles. The goal of this study was to explore floral volatile blends of different strawberry species (*Fragaria x ananassa* and *Fragaria vesca*) to identify compounds that might be used to improve the attractiveness of existing lures for SBW. Floral emissions of *F. x a.* varieties Sonata, Beltran, Korona, and of *F. vesca*, were collected by both solid-phase microextraction (SPME) and dynamic headspace sampling on Tenax. Analysis by gas chromatography/mass spectrometry showed the floral volatiles of *F. x ananassa.* and *F. vesca* were dominated by aromatic compounds and terpenoids, with 4-methoxybenzaldehyde (*p*-anisaldehyde) and α-muurolene the major compounds produced by the two species, respectively. Multi-dimensional scaling analyses separated the blends of the two species and explained differences between *F. vesca* genotypes and, to some degree, variation between *F. x ananassa* varieties In two-choice behavioral tests, SBW preferred odors of flowering strawberry plants to those of non-flowering plants, but weevils did not discriminate between odors from *F. x ananassa* and *F. vesca* flowering plants. Adding blends of six synthetic flower volatiles to non-flowering plants of both species increased the preference of SBW for these over the plants alone. When added individually to non-flowering plants, none of the components increased the preference of SBW, indicating a synergistic effect. However, SBW responded to 1,4-dimethoxybenzene, a major component of volatiles from *F. viridis*, previously found to synergize the attractiveness of the SBW aggregation pheromone in field studies.

## Introduction

The strawberry blossom weevil (SBW), *Anthonomus rubi* Herbst, (Coleoptera, Curculionidae) is an oligophagous species that feeds and reproduces on rosaceous plants (Popov 1996). Among its hosts are strawberry, *Fragaria* spp., raspberry, *Rubus idaeus* L., blackberry, *Rubus* spp. and rose, *Rosa* spp. (Hill 1987; Popov 1996). Early in spring, adults move to strawberry and raspberry from overwintering shelters, both inside the cropping area and from perimeter wild host plants (Alford 1984). There, they feed on foliage, flower buds and open flowers. The female weevil usually oviposits a single egg in an unopened bud (Aasen et al. 2004; Jary 1931; Leska 1965) before severing the bud petiole, either partially or completely, preventing further development of the bud. The oviposition period lasts 1–2 months, during which more than 150 buds can be destroyed per female (Easterbrook et al. 2003). The larva develops and pupates inside the withered bud and emerges in late summer. After emergence, the young adult feeds on foliage for a few weeks before moving to an overwintering site (Hill 1987).

The weevil is a serious pest of cultivated strawberry (*Fragaria x ananassa*) throughout Europe (Cross et al. 2001), causing bud damage to 5–90% of the crop (Aasen and Trandem 2006; Cross et al. 2001; Labanowska 2004; Leska 1965; Kovanci et al. 2005; Krauß et al. 2014; Popov 1995; Svensson 2002). Resistance to pyrethroid insecticides makes effective control of SBW challenging (Aasen and Trandem 2006).

For many insects, pheromones are key cues in mate finding (Yew and Chung 2015), and can play an important role in integrated pest control programs (Suckling et al. 2014). The male-produced aggregation pheromone of SBW was identified as a blend of grandlure I, grandlure II and lavandulol in a 1:4:1 ratio by Innocenzi et al. (2001). This pheromone blend is attractive to both sexes and is used as a lure in commercial monitoring of SBW (Cross et al. 2006a, b). However, recent data suggested that, in order to control SBW populations, more effective lure formulations are needed (Baroffio et al. 2018). For a number of pests, the attractiveness of a pheromone can be enhanced by host plant volatiles (Reddy and Guerrero 2004). This has been demonstrated for other weevils of the genus *Anthonomus* (Dickens 1989; Muniz-Merino et al. 2014). Furthermore, it has been shown that combining a floral volatile, 1,4-dimethoxybenzene, with the aggregation pheromone of *A. rubi*, gives improved trap catches (Wibe et al. 2014). This compound is now included in commercially available lures.

The objective of this study was to identify other host-plant compound(s) that might be combined with the SBW aggregation pheromone to improve attraction of SBW. We aimed: (i) to determine whether SBW is able to discriminate between volatiles from flowering versus non-flowering strawberry plants of two species, *Fragaria x ananassa* and *F. vesca*; (ii) to determine whether SBW prefers volatiles of flowering *F. x ananassa* over those of flowering *F. vesca*; (iii) to identify components of the volatile floral blends of *F. x ananassa* and *F. vesca*; and (iv) to determine whether selected components of these floral volatiles elicit behavioral responses by SBW in a laboratory bioassay when tested individually or in blends.

## Methods and Materials

### Insects and Plants

Adult SBW were collected from strawberry fields in SE Norway (N59.66, E10.69) in mid-May and transported to the laboratory at the Royal Institute of Technology, Stockholm. Both sexes were kept together on potted plants of non-flowering *Fragaria x ananassa* Duchesne (Rosales: Rosaceae), variety Sonata, enclosed in 400 cm^3^ plastic cups covered with nylon mesh. A moistened piece of cotton at the bottom of the cup served as a water source. The day before experiments, weevils were sexed and kept individually in plastic cups without plants. Sex was determined by the presence of a thorn on each intermediary coxa of male weevils (Innocenzi et al. 2002).

*Fragaria x ananassa* plants of varieties Sonata, Beltran and Korona were obtained from the Plantagen Sweden stores. Wild strawberry, ***Fragaria vesca*** L. (Rosales: Rosaceae) plants were obtained from the strawberry genotype collection held at the Ecology Department, Swedish Agricultural University, Uppsala, Sweden. Location of the nine genotypes (I-IX) was the following: I **–** N59^o^59.287, E17^o^25.578; II **–** N59^o^54.932, E17^o^08.646; III **–** N59^o^54.632, E17^o^22.775; IV **–** N59^o^53.858, E17^o^29.500; V **–** N59^o^45.729, E17^o^20.524; VI **–** N59^o^40.672, E17^o^17.660; VII **–** N59^o^31.674, E17^o^19.662; VIII **–** N59^o^21.411, E18^o^17.118; IX **–** N59^o^21.412, E18^o^17.118. The plants were kept under laboratory conditions: 16:8 h L:D photoperiod and ~ 18 °C. A 1000 W daylight lamp (type DRF, for use in greenhouses) was used as the light source.

### Chemicals

Benzaldehyde (> 98% chemical purity), 4-methoxybenzaldehyde (*p*-anisaldehyde; > 98% chemical purity), methyl salicylate (> 98% chemical purity) and benzyl alcohol (> 99% chemical purity) were purchased from Alfa Aesar (Ward Hill, Massachusetts, USA). (±)-Limonene (> 98% chemical purity), decanal (> 98% chemical purity), 1,4-dimethoxybenzene (> 99% chemical purity) and pentadecane (> 99% chemical purity) were obtained from Sigma-Aldrich AB (Stockholm, Sweden), while α-muurolene (95% chemical purity) and analytical standards in Table 1 were available from the Ecological Chemistry group (Stockholm, Sweden). Diethyl ether (redistilled, 99.9%) and cyclohexane (99.9%) were purchased from Carlo Erba Reagents (Val de Reuil, France).

**Table 1:** Composition of odor blends from flowers of *Fragaria ananasa* cultivars Sonata, Beltran and Corona and *F. vesca* collected by solid phase microextraction, and previously reported olfactory activity of *Anthonomus rubi* to the compounds

					Mean TIC count/g dry weight/h ± SE (× 10 million)^e^				
No.	Of Compound	GR^a^	RI^b^	ID^c^	*F. a.* Sonata	*F. a.* Beltran	*F. a.* Corona	*F. vesca*	OA^f^
1	α-Pinene	ΜΤ	1017	RC	1 ± 1 b^g^	3 ± 1 b	0	99 ± 19 a	‡
2	β-Pinene	ΜΤ	1105	RC	0	tr	0	34 ± 23	‡
3	3-Carene	ΜΤ	1151	RC	1 ± 1 b	2 ± 1 b	0	18 ± 7 a	‡
4	Limonene	ΜΤ	1196	RC	19 ± 6 b	19 ± 3 b	33 ± 8 b	159 ± 27 a	§
5	β-Phellandrene	MT	1207	L, RI	tr	tr	2 ± 2 a	9 ± 4 a	§
6	(*Z*)-β-Ocimene	MT	1239	RC	0	0	0	tr	§
7	(*E*)-β-Ocimene	MT	1249	RC	0	9 ± 9 a	0	24 ± 14 a	§
8	*p*-Cymene	ARMT	1260	RC	tr	tr	tr	8 ± 5 a	
9	Hexyl acetate	E	1269	RC	tr	2 ± 1 b	1 ± 1 b	10 ± 3 a	‡
10	1-Ethyl-2-methyl-benzene	AR	1271	RC	tr	2 ± 1 b	0	25 ± 5 a	
11	Octanal	AL	1283	RC	9 ± 1 c	18 ± 4 bc	38 ± 17 b	108 ± 34 a	
12	(Z)-3-Hexen-1-yl acetate	E	1312	RC	43 ± 30 ab	12 ± 4 b	53 ± 15 a	80 ± 12 a	‡
13	Methoxybenzene	AR	1330	RC	10 ± 4 ab	2 ± 1 b	11 ± 3 a	0	
14	6-Methyl-5-hepten-2-one	K	1331	RC	5 ± 1 c	12 ± 3b	31 ± 12 b	86 ± 11 a	
15	1-Hexanol	OH	1354	RC	7 ± 3 ab	1 ± 1 b	14 ± 10 ab	46 ± 26 a	‡
16	methoxymethyl-Benzene	AR	1379	RC	26 ± 19 a	2 ± 1 a	0	0	
17	(Z)-3-Hexen-1-ol	OH	1380	RC	tr	1 ± 1 b	10 ± 6 a	27 ± 7 a	‡
18	Nonanal	AL	1388	RC	42 ± 5 c	80 ± 10 b	212 ± 39 a	428 ± 80 a	
19	Copaene	ST	1483	RC	0	0	0	22 ± 14	
20	Decanal	AL	1493	RC	64 ± 10 b	171 ± 32 ab	310 ± 50 a	525 ± 134 a	
21	Benzaldehyde	AR	1501	RC	78 ± 4 ab	141 ± 55 a	40 ± 15 b	64 ± 10 ab	
22	Linalool	OMT	1534	RC	tr	0	0	0	‡
23	β-Caryophyllene	ST	1587	RC	0	0	0	25 ± 18	§
24	Methyl benzoate	AR	1602	RC	13 ± 2 a	9 ± 5 a	8 ± 1 a	12 ± 6 a	§
25	3,6,6-Trimethyl-2-norpinanone	OMT	1618	L, RI	0	0	0	11 ± 6	
26	Acetophenone	AR	1630	RC	tr	3 ± 1 a	1 ± 1 a	13 ± 13 a	
27	1-Ethenyl-4-methoxy-benzene	AR	1661	RC	3 ± 2 b	26 ± 8 a	0	0	
28	3-Ethyl-benzaldehyde	AR	1690	RC	8 ± 3 b	5 ± 2 b	6 ± 2 b	24 ± 5 a	
29	Germacrene D	ST	1696	RC	0	0	0	2 ± 1	§
30	α-Muurolene	ST	1716	RC	11 ± 2 b	10 ± 5 b	14 ± 6 b	604 ± 104 a	
31	4-Ethyl-benzaldehyde	AR	1718	RC	6 ± 2 b	10 ± 3 ab	6 ± 2 b	20 ± 5 a	
32	(*E*,*E*)-α-Farnesene	ST	1743	RC	0	2 ± 2 b	0	128 ± 34 a	
33	Unidentified 1 (sesquiterpene)	ST	1746	L, RI	tr	2 ± 2 a	0	4 ± 3 a	
34	Methyl salicylate	AR	1754	RC	96 ± 18 b	59 ± 27 b	35 ± 18 b	268 ± 32 a	§
35	TMTT^d^	HT	1801	RC	0	0	0	15 ± 8	
36	Dihydro α-ionone	TK	1802	RC	tr	12 ± 5	0	0	
37	Unidentified 2 (sesquiterpene)	ST	1847	L, RI	0	4 ± 2 a	12 ± 6 a	0	
38	Benzyl alcohol CAS#:	AR	1859	RC	310 ± 32 a	136 ± 63b	99 ± 24 b	312 ± 41 a	
39	Benzyl isovalerate	AR	1876	RC	4 ± 1 ab	22 ± 11 a	5 ± 3 ab	0	
40	2-Phenylethanol	AR	1894	RC	17 ± 2 ab	8 ± 3 b	88 ± 47 a	106 ± 48 a	
41	1,4-Butanediol	OH	1912	L, RI	0	0	0	20 ± 11	
42	1,2-Benzisothiazole	O	1931	L, RI	26 ± 14 a	6 ± 2 a	8 ± 1 a	14 ± 5 a	
43	4-Metoxy-benzaldehyde	ARE	1998	RC	1997 ± 541 a	1144 ± 433 a	1532 ± 536 a	0	
44	Methyl 2-methoxy-benzoate	ARE	2049	RC	12 ± 7 a	8 ± 4 a	10 ± 1 a	0	
45	Benzyl 2-methyl-(*E* or *Z*)-2-butenoate	ARE	2092	L, RI	30 ± 5 a	70 ± 46 a	23 ± 13 a	0	
46	Hexahydrofarnesyl acetone	TK	2120	RC	6 ± 2 b	19 ± 6 ab	14 ± 4 ab	35 ± 7 a	
47	2-Phenoxyethanol	AR	2122	RC	3 ± 2 b	tr	4 ± 1 a	tr	
48	Unidentified 3		2133		28 ± 3 a	7 ± 3 b	26 ± 8 a	0	
49	Unidentified 4	E	2240		15 ± 5 a	8 ± 6 a	25 ± 9 a	59 ± 20 a	
50	4-Methoxybenzyl ethanol	AR	2257	RC	60 ± 14 a	24 ± 6 b	34 ± 9 ab	0	
51	3,4-Dimethoxybenzaldehyde	AR	2365	RC	7 ± 5 a	3 ± 1 b	tr	0	
52	Unidentified 5		2376		16 ± 6 a	7 ± 5 a	19 ± 6 a	52 ± 13 a	
53	Unidentified 6		2480		19 ± 7 a	11 ± 7 a	32 ± 5 a	60 ± 24 a	
54	Unidentified 7		2487		49 ± 18 a	9 ± 6 a	6 ± 2 a	0	
55	Benzyl benzoate	AR	2629	RC	46 ± 8 a	136 ± 80 a	19 ± 10 a	0	
56	Unidentified 8		2690		11 ± 7 a	3 ± 3 a	12 ± 7 a	0	
57	Unidentified 9		2851		tr	tr	0	44 ± 14	

^a^GR = group of chemical compound (MT monoterpene; ARMT aromatic monoterpene; E ester; AR aromatic; AL aldehyde; K ketone; OH alcohol; OMT oxygenated monoterpene; ST sesquiterpene; HT homoterpene; TK terpene ketone; ARE aromatic ester; O other compound)

^b^RI = retention index (DB-Wax fused silica capillary column 30 m × 0.25 mm i.d., 0.25 μm film thickness)

^c^ID = identification source; RC = reference compound; RI = retention index; L = NIST and MassFinder3 libraries

^d^TMTT = (3E,7E)-4,8,12- Trimethyltrideca-1,3,5,7,11-tetraene

^e^TIC = total ion chromatogram; SE = standard error of mean; tr = trace; F. a. Sonata (*N* = 3), F. a. Beltran (N = 4), F. a. Corona (N = 4), and F. vesca (N = 9)

^f^OA = olfactory activity reported in *A. rubi*; § Bichão et al. 2005a, ‡ Bichão et al. 2005b

^g^The means indicated by the same letter in each row are not different (nonparametric Conover-Iman test, *P* < 0.05)

### Sampling and Analysis of Floral Volatiles

We used solid-phase microextraction (SPME; Rout et al. 2012) to sample volatiles of three varieties of *F. x ananassa* and flowers of different genotypes of *F. vesca* using the same fiber type, headspace volume, and temperature during sampling. An internal standard was used to check for saturation of fibers. This is a sensitive technique and provided quantitative data for statistical comparisons among varieties and species. Even so, comparison of quantities of different compounds in the same sample is not possible without use of labelled standards, because of different affinities of the fiber for compounds and different vapor pressures of compounds (SPME guidelines 2020). Thus, dynamic headspace sampling with an internal standard was also used for more reliable quantification of compounds and release rates.

SPME sampling was carried out with polydimethylsiloxane-divinylbenzene-coated fibers (65 μm; Supelco, Sigma-Aldrich group, PA, USA) (Vas and Vekey 2004), desorbed at 250 °C for 2 min in a gas chromatograph (GC) injector prior to sampling. A single, ready-to-flower bud from a potted strawberry plant was placed in a glass chamber (30 cm^3^) through an opening at the bottom, along with a filter paper (1 cm^2^) treated with 100 ng of pentadecane per 10 μl of cyclohexane as internal standard. The opening was carefully sealed with aluminum foil. After the bud had opened, the SPME fiber was introduced close to the flower through a second opening in the chamber. Sampling of volatiles was carried out from 08.00 until 18.00 h covering the main period of emission (Ceuppens et al. 2015). After collection, the fiber was removed and desorbed directly in the injector of the gas chromatograph/mass spectrometer (GC/MS). Volatiles were collected from individual flowers of *F. ananassa* variety Sonata (*N* = 3), *F. ananassa* variety Beltran (*N* = 4), *F. ananassa* variety Corona (*N* = 4), and *F. vesca* (*N* = 9).

Dynamic headspace sampling (Millar and Haynes 1998) was carried out on individual flowers of *F. ananassa* variety Sonata (*N* = 3) and *F. vesca* (*N* = 3). A single, ready-to-flower bud was placed in the type of glass jar used for SPME sampling. Charcoal-purified and humidified air (50 ml.min^−1^) was supplied by a diaphragm vacuum pump (NMP 830 KNDCB; KNF Neuberger Inc., Freiburg, Germany), and pulled through a glass collection tube containing Tenax TA adsorbent (50 mg; 60/80 mesh; Sigma-Aldrich AB, Sweden) by a second pump. Volatiles were collected from 08.00–18.00 h as above. After sampling, traps were extracted with 250 μl of redistilled diethyl ether, and 20 ng of pentadecane in cyclohexane was added as internal standard. Samples were concentrated under a gentle flow of nitrogen and analyzed on the same day as collected.

After sampling, flowers were detached from the peduncle, placed in a glass beaker and kept in a thermostat at 60 °C for 72 h for determination of dry weight. For both sampling techniques, volatiles were also collected from empty glass chambers.

Samples were analyzed using a Varian 3400 GC coupled to a Finnigan SSQ 7000 MS (Thermo-Fisher Scientific, USA). A DB-Wax fused silica capillary column (30 m length, 0.25 mm id, 0.25 μm film thickness; Supelco-Sigma-Aldrich group, USA) was used. The column oven was programmed from 40 °C for 3 min, then at 4 °C.min^−1^ to 200 °C, then at 10 °C.min^−1^ to 230 °C, and held for 9 min. The split/splitless injector temperature was 225 °C and the splitless period lasted for 60 s. Helium was used as carrier gas with an inlet pressure of 70 kPa. The transfer line temperature was 235 °C. Electron ionization mass spectra were determined at 70 eV with an ion source at 150 °C. Chromatographic profiles of volatiles were compared and compounds in large amounts, relative to those in blank samples, identified by comparison of mass spectral data and retention indices with synthetic standards (Table 2), along with data from NIST version 2.0 mass spectral search program (National Institute of Standards and Technology, USA). Relative amounts of compounds were determined as areas under chromatographic peaks. Absolute amounts of compounds trapped by aeration were quantified by applying standard calibration curves derived from pentadecane at 0.5 ng, 1 ng, 10 ng and 50 ng.

**Table 2:** Composition of volatiles from flowers of *Fragaria ananasa* cultivar Sonata and *F. vesca* flowers collected by dynamic headspace technique

					Mean rate ± SE (ng/g dry weight/h)^e^	
No	Compound	GR^a^	RI^b^	ID^c^	*F. a. Sonata*	*F. vesca*
1	α-Pinene	MT	1017	RC	0.5 ± 0.29	4.4 ± 3.07def^f^
	Hexanal	AL	1076	RC	0.3 ± 0.15	0
2	β-Pinene	ΜΤ	1105	RC	0.2 ± 0.06	0
3	3-Carene	MT	1151	RC	0.3 ± 0.12	0
	Heptanal	AL	1180	RC	0.3 ± 0.02	2.4 ± 1.8f
4	Limonene	MT	1196	RC	1.6 ± 0.52d	11.9 ± 1.46bcd
	Propylbenzene	AR	1198	RC	0.3 ± 0.20	0
5	β-Phellandrene	ΜΤ	1207	L, RI	0.1 ± 0.04	0
	(*E*)-2-Hexenal	AL	1208	RC	0.1 ± 0.05	3.5 ± 2.44ef
6	(*Z*)-β-Ocimene	MT	1239	RC	tr	0
7	(*E*)-β-Ocimene	MT	1249	RC	tr	3.0 ± 1.2ef
8	*p*-Cymene	ARMT	1260	RC	tr	0
10	Hexyl acetate	E	1269	RC	0.1 ± 0.06	0
11	Octanal	AL	1284	RC	3.7 ± 0.60 cd	6.8 ± 0.45de
12	(*Z*)-3-Hexen-1-yl acetate	E	1312	RC	2.1 ± 1.07d	9.1 ± 1.91bcd
13	Methoxybenzene	AR	1331	RC	0.7 ± 0.55	0
14	6-Methyl-5-hepten-2-one	K	1332	RC	9.7 ± 2.16	tr
15	1-Hexanol	OH	1355	RC	2.1 ± 0.25	0
16	Methoxymethyl-benzene	AR	1380	RC	0.3 ± 0.12	0
17	(Z)-3-Hexen-1-ol	OH	1381	RC	0.8 ± 0.26	0
18	Nonanal	AL	1389	RC	13.0 ± 1.63b	9.7 ± 0.71bcd
19	α-Copaene	ST	1484	RC	tr	0
20	Decanal	AL	1493	RC	18.9 ± 7.78ab	8.9 ± 1.76bcd
21	Benzaldehyde	AR	1501	RC	9.6 ± 0.76b	14.5 ± 2.58ab
22	Linalool	OMT	1534	RC	0.2 ± 0.08	7.3 ± 1.71cde
23	β-Caryophyllene	ST	1587	RC	0.0	2.5 ± 0.64f
	Undecanal	AL	1597	RC	1.5 ± 0.4d	0
24	Methyl benzoate	AR	1602	RC	2.1 ± 0.41d	0
26	Acetophenone	K	1630	RC	0.2 ± 0.14	8.2 ± 2.21bcd
27	1-Ethenyl-4-methoxybenzene	AR	1661	RC	1.0 ± 0.11	0
29	Germacrene D	ST	1696	RC	0.4 ± 0.27	1.4 ± 0.73f
	Unidentified 1 (sesquiterpene)	ST	1715	RC	0.1 ± 0.04	0
30	α-Muurolene	ST	1716	RC	0.1 ± 0.05	18.5 ± 1.79a
32	(*E*,*E*)-α-Farnesene	ST	1743	RC	1.2 ± 0.26d	0
34	Methyl salicylate	AR	1754	RC	2.6 ± 0.41c	9.0 ± 1.47bcd
35	TMTT^d^	HT	1801	RC	0.5 ± 0.22	4.1 ± 0.86ef
	Unidentified 2 (sesquiterpene)	OST	1847	L, RI	11.9 ± 7.55abc	0
38	Benzyl alcohol	AR	1859	RC	11.0 ± 5.18ab	10.8 ± 2.35bc
39	Benzyl isovalerate	AR	1875	RC	tr	0
40	2-Phenylethanol	AR	1893	RC	3.2 ± 2.79 cd	0
42	1,2-Benzisothiazole	O	1930	L, RI	tr	0
43	4-Methoxybenzaldehyde	AR	1997	RC	23.0 ± 2.37a	0
44	Methyl 2-methoxybenzoate	ARE	2048	RC	tr	0
45	Benzyl 2-methyl-(*E* or *Z*)-2-butenoate	ARE	2091	L,RI	tr	0
	(*Z*)-3-Hexen-1-ol benzoate	E	2103	RC	tr	0
46	Hexahydrofarnesyl acetone	TK	2119	RC	tr	0
	Unidentified 3		2132		0.6 ± 0.45	0
	Unidentified 4		2239		0.1 ± 0.4	0
50	4-Methoxybenzyl alcohol	AR	2256	RC	tr	0
55	Benzyl benzoate	AR	2629	RC	0.2 ± 0.12	0

^a^GR = group of chemical compound (MT = monoterpene; AL = aldehyde; AR = aromatic; ARMT = aromatic monoterpene; E = ester; HT = homoterpene; K = ketone; O = other compound; OH = alcohol; OMT = oxygenated monoterpene; ST = sesquiterpene; TK = terpene ketone

^b^RI = retention index (DB-Wax fused silica capillary column 30 m × 0.25 mm i.d., 0.25 μm film thickness)

^c^ID = identification source; RC = reference compound; RI = retention index; L = NIST and MassFinder3 libraries

^d^TMTT = (3*E*,7*E*)-4,8,12- Trimethyltrideca-1,3,5,7,11-tetraene

^e^SE = standard error of mean; tr = trace; *F. a.* Sonata (N = 3) and *F. vesca* (N = 3)

^f^The means indicated by the same letter in each column are not different (nonparametric Conover-Iman test, P < 0.05, calculated for the compounds with amount exceeding 1 ng)

### Behavioral Tests

Behavioral responses of SBW to natural and synthetic odors were tested in two-choice olfactometers. The test area was illuminated with a quartz metal halide lamp (HPI-T Plus 400 W; Philips, Amsterdam, the Netherlands) placed 180 cm above the olfactometers. The olfactometers comprised three layers of acrylic plastic (each layer 0.5 cm thick) sandwiched together with an arena cut out in the middle layer and consisting of a central zone (2 × 2.5 cm) with two tapered arm zones (4 cm length from the air inlet to the central zone and 0.4 cm to 2.5 cm width at the inlet and the central zone, respectively) (Hambäck et al. 2003) (Fig. 5a). One vacuum diaphragm pump delivered stimuli in purified and humidified air to the arms of the olfactometer and another pump was connected via Teflon tubes to the top so as to withdraw air at ca. 3 ml.sec^−1^. Four olfactometer trials could be run simultaneously, testing an odorant of the same type. Before each trial, a weevil was allowed to acclimatize inside the olfactometer for 3 min. During delivery of the stimulus, the weevil’s position in the arena was noted at 30 s intervals for 15 min, giving a total of 30 recordings for each individual weevil. The responsiveness of a SBW was assessed by calculating the percentage of records in each arm. Each weevil was only used once in an experiment, and weevils that were inactive in the central zone for more than 5 min during trials were excluded from the analysis. Between trials, the olfactometers were washed with water and a mild detergent.

In the first experiment, weevil preference to the olfactometer arms without olfactory cues was tested to confirm lack of bias in the olfactometer. In the second experiment, weevil responses to odors from flowering versus non-flowering strawberry plants were evaluated. For the non-flowering plants, buds or flowers were removed 2 d prior to the experiment. A single potted flowering and non-flowering *F. x ananassa* plant were placed separately in a polyester cooking bag (25 × 40 cm; Toppits, Minden, Germany), while 4–5 potted flowering and non-flowering *F. vesca* plants (collected at N59^o^21.412, E18^o^17.118) of total leaf area approximately equal (estimated by visual evaluation) to that of the *F. x ananassa* plant were placed together in another polyester cooking bag. One *F. x a.* var. Sonata flower corresponded to four *F. vesca* flowers, based on dry weight. The pots were covered with aluminum foil in order to minimize soil volatiles in the headspace. Purified and humidified air was delivered at a ca. 12 ml.sec^−1^ from the bottom of the bag, and volatiles were collected at the top of the bag.

In the third experiment we tested preference for flowering *F. vesca* versus flowering *F. x a.* var. Sonata plants, using five wild strawberry plants with eleven flowers and one garden strawberry plant with three flowers, respectively.

In the fourth experiment, the responses of SBW to synthetic chemicals added to non-flowering plants were tested against plants alone. Compounds were dissolved in cyclohexane and 10 μl of solution applied to 2 cm^2^ of filter paper in a glass vial (10 cm^3^) positioned close to the plant. Single compounds were tested at a dose of 100 ng. Two six-component blends of synthetic compounds were made to represent floral bouquets of *F. x ananassa* var. Sonata (FAS) and *F. vesca* (FV), respectively*.* The FAS blend comprised 4-methoxybenzaldehyde, benzaldehyde, benzyl alcohol, methyl salicylate, limonene and decanal at loadings of 200, 15, 51, 20, 9 and 40 ng, respectively. The FV blend comprised α-muurolene, benzaldehyde, benzyl alcohol, methyl salicylate, limonene and decanal at loadings of 30, 25, 45, 60, 70 and 80 ng, respectively. The loading ratios and amounts of synthetic odorants were selected to emit profiles similar to those of strawberry flowers. Five components in these two synthetic blends were common, consistent with the observation that SBW showed no preference for flowering plants of either species. All five compounds were previously shown to elicit electrophysiological responses from the antennae of SBW by Bichão et al. (2005a). Furthermore, these compounds represent different classes of floral volatiles and would be economically feasible for practical control use. In addition, the FAS blend included 4-methoxybenzaldehyde, the major component in floral volatiles from cultivated strawberry, while the FV blend contained α-muurolene, the major component in volatiles from wild strawberry.

In the fifth experiment, the responses of SBW to 4-methoxybenzaldehyde added to non-flowering *F x ananassa* var. Sonata plants at doses of 10 ng, 100 ng and 1000 ng were investigated.

### Data Analysis

To monitor saturation of SPME fibers, the amounts of pentadecane adsorbed were compared by Mann Whitney U test, using Statistica software version 6.0.

To compare amounts of floral volatiles between *F. x ananassa* varieties and *F. vesca*, data were analyzed by Kruskal-Wallis a non-parametric test followed by a Conover-Iman test using R (version 4.0.2) and Rstudio (version 1.3.959).

To assess and visualize associations among odor blends of strawberry flowers sampled by SPME, a multidimensional scaling (MDS) analysis with a Bray-Curtis index was performed on absolute amounts, expressed as areas under chromatographic peaks, using R (version 4.0.2) and Rstudio (version 1.3.959), with the metaMDS function in the vegan package (version 2.5–6). The results were visualized using ggplot2 (version 3.3.2). Prior to analysis, the data were square root transformed. Amounts of volatiles were also used to show degree of similarity of odor bouquets between *F. x ananassa* varieties and *F. vesca* by cluster analysis, based on Euclidian distance using Statistica software version 6.0.

The behavioral responses of SBW in two-choice olfactometer tests were analyzed by nonparametric Wilcoxon matched-pairs signed-ranks test using the Statistica software version 6.0.

## Results

### Chemical Composition and Variation of Strawberry Floral Odor Blends

Using SPME, 46, 39 and 49 compounds were detected in floral volatiles from *F. x ananassa* varieties Sonata, Beltran, and Korona, respectively. In the flower headspace of *F. vesca,* 41 compounds were detected (Table 1). Monitoring saturation of SPME fibers by adding 100 ng of pentadecane as internal standard to the samples revealed no differences in amount of pentadecane trapped on the fibers in blank samples (empty glass jars; median TIC count 17,934,247) versus *F. vesca* flowers (median TIC count 15,258,935) and versus flowers of *F. x ananassa* variety Sonata (median TIC count 11,565,433) (Mann Whitney U test, *N* = 5, *P* = 0.222 and *N* = 3, *P* = 0.071, respectively).

**Fig. 1:**
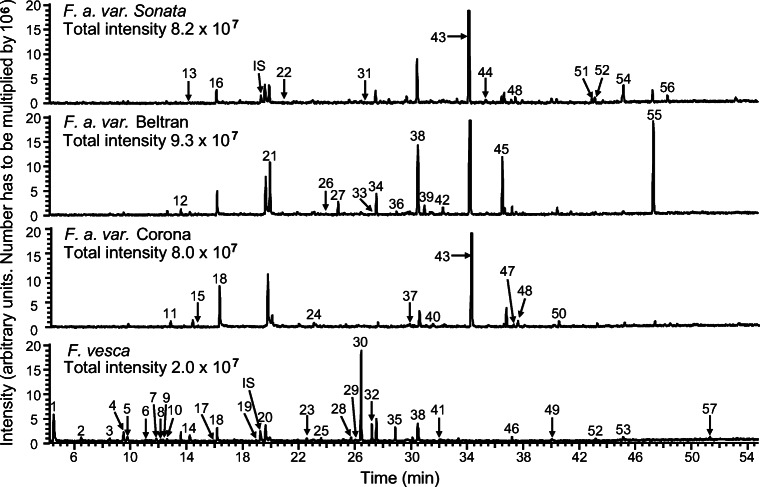
Total ion chromatograms from gas chromatography/mass spectrometry analyses of floral odors collected by solid phase microextraction headspace sampling of a single flower of *Fragaria x ananassa* varieties Sonata, Beltran, Korona, and *F. vesca*. (DB-Wax fused silica capillary column; numbered chromatographic peaks are listed in Tables 1 and 2; IS pentadecane internal standard)

Using the dynamic headspace sampling technique, 49 compounds were detected in floral volatiles collected from *F. x ananassa* variety Sonata and 18 compounds from *F. vesca* (Table 2). For both techniques, all samples were from single flowers. GC/MS analyses revealed that floral volatile blends of both species were dominated by aromatic compounds and terpenoids. From the flowers of *F. x ananassa,* 4-methoxybenzaldehyde (*p*-anisaldehyde) was collected in the largest quantity, and from *F. vesca,* α-muurolene (Fig. 1, Tables 1 and 2).

**Fig. 2:**
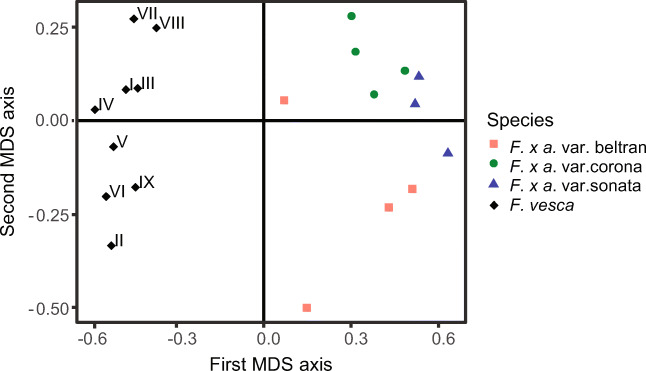
Score plot of odor blends sampled by solid phase microextraction of headspace of single flowers of *Fragaria x ananassa* varieties Sonata, Beltran, Korona, and *Fragaria vesca* potted plants. Roman numerals represent genotype of *F. vesca* plants; MDS = multidimensional scaling

Multidimensional scaling (MDS) analysis, using data from SPME samplings, showed that odor blends released from *F. x ananassa* and *F. vesca* flowers were separated from each other (Fig. 2). The first MDS axis explained separation between specimens of *F. x ananassa* and *F. vesca* species. Twenty four compounds had significantly higher amounts per gram of dry flower weight released per hour for *F. vesca* than for *F. x ananassa* flower samples (Table 1). The odor blends of *F. vesca* flowers were characterized by seven unique compounds, including six terpenoids [α-copaene (19), β-caryophyllene (23), 3,6,6-trimethyl-2-norpinanone (25), germacrene D (29), (3E,7E)-4,8,12- rimethyltrideca-1,3,5,7,11-tetraene (TMTT) (35) and β-phellandrene (5)], as well as one alcohol [1,4-butanediol (41)]. Of the 24 compounds, 15 were terpenoids, three aromatics, three aldehydes, and one each of a ketone, ester and unidentified compound (Fig. 2, Table 1). Floral volatile blends from *F. x ananassa* contained 17 unique compounds, including 10 aromatics, two terpenoids, one ketone and four unidentified compounds (Table 1).

**Fig. 3:**
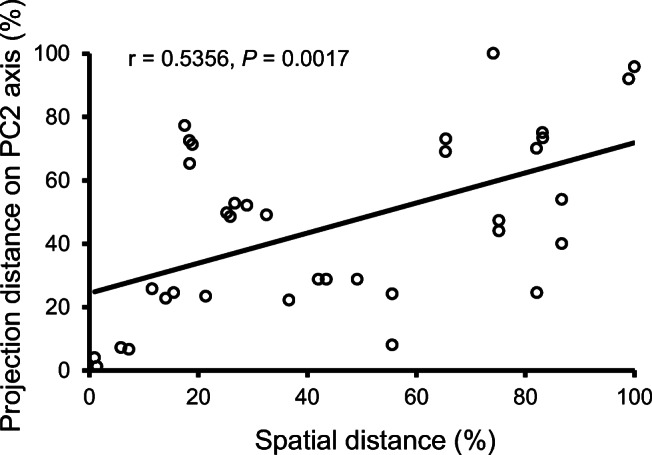
Relationship between geographic separations of *Fragaria vesca* genotypes in the field and differences in floral odor blends expressed as projection distances of blends on the second axis in the multidimensional scaling score plot in Fig. 2. Both spatial and floral blend projection distances were transformed to percentage scale assigning the largest distance to 100%. The largest spatial distance between origins of two genotypes was 95 km

**Fig. 4:**
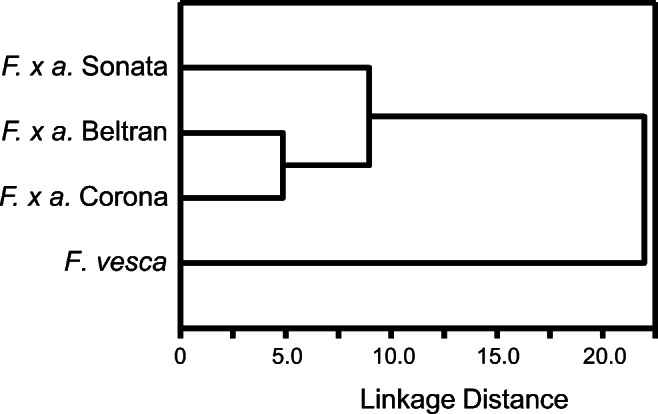
Dendrogram of odor blends sampled by solid phase microextraction of headspace of single flowers of *Fragaria x ananassa* varieties Sonata, Beltran, Korona, and *Fragaria vesca*. Dendrogram was obtained by cluster analysis based on Euclidian distance. Numbers on x axis have to be multiplied by 10^9^

The second MDS axis explained differences between *F. vesca* genotypes and some of the variation between *F. x ananassa* varieties (Fig. 2). A significant correlation (r = 0.5356, *P* = 0.017) (Fig. 3) was found between geographical separation of *F. vesca* genotypes and differences in floral odor blends, expressed as projection distances of blends on the second MDS axis in Fig. 2. Flowers of variety Sonata had the odor blend most distinct from the other two varieties of *F. x ananassa* (Fig. 4), and were characterized by large amounts of benzyl alcohol (38), 3,4-dimethoxybenzaldehyde (51) and linalool (22). Odor blends of variety Korona were distinguished by larger amounts of β-phellandrene (5), (*Z*)-3-hexen-1-ol (17) and nonanal (18) and the absence of eight compounds compared to the other two cultivars (Table 1). (*E*)-β-Ocimene (7), 1-ethenyl-4-methoxybenzene (27), (*E*,*E*)-α-farnesene (32), an unidentified sesquiterpene (37) and β-pinene (29) were present in larger quantities in the odor blends of variety Beltran.

### Olfactory Preferences of *Anthonomus rubi*

**Fig. 5:**
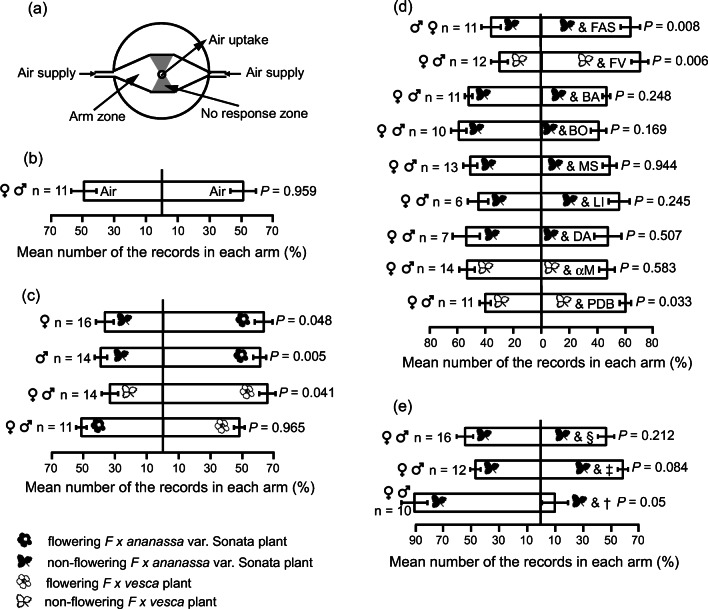
**a** Schematic of two-choice olfactometer. Behavioral responses of *Anthonomus rubi* weevils in two-choice olfactometer to (**b**) air, **c** odors of flowering and non-flowering *Fragaria x ananassa* variety Sonata and *F. vesca* plants; **d** mixtures or single synthetic compounds, found in floral odors of *F. x a.* var. Sonata, *F. vesca* and *Fragaria viridis;* **e** three doses of 4-methoxybenzaldehyde. Vertical bars are SEM; n = number of weevils tested; FAS is the six-component blend of 4-methoxybenzaldehyde, benzaldehyde (BA), benzyl alcohol (BO), methyl salicylate (MS), limonene (LI) and decanal (DA); FV is the six-component blend of α-muurolene (αM), BA, BO, MS, LI and DA; PDB = 1,4-dimethoxybenzene. §, ‡ and † represent 4-methoxy-benzaldehyde at doses of 10, 100 and 1000 ng, respectively; Data were analyzed by nonparametric Wilcoxon matched-pairs signed-ranks test

SBW showed no preference for either olfactometer arm when no olfactory cue was present, showing no inherent bias in the bioassay (Fig. 5b). When given the choice between odors of flowering *F. x ananassa* var. Sonata plants and odors from non-flowering plants, both male and female SBW preferred those from the flowering plant (*N* = 16, *Z* = 2.811, *P* = 0.005 and *N* = 14, *Z* = 1.977, *P* = 0.048, respectively) (Fig. 5c). In further experiments, SBW were not separated by sex.

When testing SBW preference to odors of flowering versus non-flowering *F. vesca*, more weevils chose odors of flowering plants (*N* = 14, *Z* = 2.068, *P* = 0.041). No preference was observed between odors of flowering *F. x a.* var. Sonata and *F. vesca* plants (*N* = 11, *Z* = 0.044, *P* = 0.965) (Fig. 5c).

SBW preferred non-flowering *F. x a.* var. Sonata strawberry plants with the FAS synthetic odor blend (4-methoxybenzaldehyde, benzaldehyde, benzyl alcohol, methyl salicylate, limonene and decanal) compared to non-flowering plants alone (*N* = 11, Z = 2.667, *P* = 0.008). Similarly, SBW preferred non-flowering *F. vesca* plants with the FV blend (α-muurolene, benzaldehyde, benzyl alcohol, methyl salicylate, limonene and decanal) over flowering plants alone (*N* = 12, *Z* = 2.746, *P* = 0.006) (Fig. 5d).

None of the compounds in the two blends when added individually at 100 ng to non-flowering strawberry plants increased or decreased preference of SBW over non-flowering plants alone (benzaldehyde *N* = 11, Z = 1.156, *P* = 0.248; benzyl alcohol *N* = 10, Z = 1.376, *P* = 0.169; methyl salicylate *N* = 13, Z = 0.069, *P* = 0.944; α-muurolene *N* = 14, Z = 0.549, *P* = 0.583; limonene *N* = 6, Z = 1.531, *P* = 0.245, and decanal *N* = 7, Z = 1.726, *P* = 0.507) (Fig. 5d). Data on the effects of limonene and decanal should be considered preliminary due to the low number of replicates.

In the final bioassay experiment, SBW did not discriminate between odors released from non-flowering *F. x a.* var. Sonata strawberry plants and those from non-flowering plants of the same variety with added 4-methoxybenzaldehyde at 10 ng or 100 ng doses (*N* = 16, Z = 1.172, *P* = 0.241 and *N* = 12, Z = 1.579, *P* = 0.114, respectively). When the dose was increased to 1000 ng, weevils preferred the side with the non-flowering plant alone (*N* = 10, Z = 2.803, *P* = 0.005) (Fig. 5e).

## Discussion

### Chemical Composition and Variation of Strawberry Floral Odor Blends

The composition of floral volatile emissions from several species of strawberry have been reported previously, including *Fragaria x ananassa* (Bichão et al. 2005a; Ceuppens et al. 2015; Hamilton-Kemp et al. 1990, 1993; Klatt et al. 2013), *F. virginiana* Duchesne (Ashman et al. 2005), *F. vesca* (Blažytė-Čereškienė et al. 2017; Wibe et al. 2014) and *F. viridis* Duchesne (Blažytė-Čereškienė et al. 2017). In those studies, static and dynamic headspace collections as well as hydro-distillation techniques were used to sample floral compounds from cut and intact flowers, making it difficult to compare data. The amounts of volatiles produced by individual strawberry flowers are very small; hence, we used SPME to sample volatile profiles under standardized conditions. This provided a highly sensitive technique able to detect more compounds than in the above studies, and also provided quantitative data for comparisons of varieties and species. Dynamic headspace sampling with an internal standard was also used for more reliable quantification of compounds and release rates.

We found that 4-methoxybenzaldehyde (*p*-anisaldehyde) was the major constituent of the floral volatile blends released by all three *F. x ananassa* varieties. This contrasts with previous reports in which benzaldehyde was present in the largest amount in volatile emissions of *F. x ananassa* varieties Darselect, Honeoye (Klatt et al. 2013) and Korona (Bichão et al. 2005a). (*E*,*E*)-α-Farnesene and limonene were reported as the major component of floral emissions of the variety Sonata by Klatt et al. (2013) and Ceuppens et al. (2015), respectively. In our study, these compounds were present at lower amounts in Sonata flowers. The reason for the quantitative differences in these studies is unknown.

In our study, α-muurolene was the major component in volatiles from flowers of *F. vesca*. 1,4-Dimethoxybenzene was reported as the major constituent of *F. vesca* floral volatile emissions by Wibe et al. (2014), contributing 96.6% of the total amount. However, neither we nor Blažytė-Čereškienė et al. (2017) detected this compound in the blends released by *F. vesca* flowers. 1,4-Dimethoxybenzene was one of the major components present in samples of *F. viridis* flowers (Blažytė-Čereškienė et al. 2017), suggesting that Wibe et al. (2014) worked with other strawberry species or hybrids, rather than *F. vesca*.

The SPME data were used to carry out MDS analyses, which separated volatile blends released from *F. x ananassa* and *F. vesca* flowers. The analyses also explained differences between *F. vesca* genotypes, and partly explained the variation among the volatile blends from the three varieties of *F. x ananassa* varieties. Flowers of variety Sonata had the most distinct odor blend.

Single cell recordings had previously revealed 58 identified and a few unidentified compounds that elicited responses of SBW olfactory receptors (Bichão et al. 2005a, b). We detected 16 of these compounds in strawberry floral volatile emissions.

Despite the differences in composition of blends of volatiles from the flowers of different species and varieties of strawberry, there were at least 13 common compounds. Five of the most abundant were benzaldehyde, benzyl alcohol, methyl salicylate, limonene and decanal. These were combined with 4-methoxybenzaldehyde and α-muurolene to give blends representative of *F. x ananassa* (FAS) and *F. vesca* (FV), respectively, for testing in bioassays. All these compounds elicited electrophysiological responses from antennae of SBW (Bichão et al. 2005a).

### Olfactory Preferences of *Anthonomus rubi*

Our data showed that SBW preferred odors of flowering strawberry over those of non-flowering plants. This was somewhat surprising, as SBW females oviposit in flower buds prior to opening. Possibly, floral volatiles are detected from the bud before opening, or weevils are attracted to the area by neighboring flowers which have already opened. A similar preference for odors released from flowering over non-flowering hosts was also reported for cranberry weevils, *Anthonomus musculus* Say (Szendrei et al. 2009), which have oviposition and feeding strategies similar to SBW. However, only female *A. musculus* showed preference to odor blends released by blueberry flowers over those of flower buds (Szendrei et al. 2009). In our study, we did not detect any sex differences in preference.

Addition of a six-component blends of chemicals to non-flowering plants of both cultivated *F. x ananassa* var. Sonata and wild species *F. vesca*, increased attractiveness to SBW, relative to non-flowering plants alone. The FAS blend mimicking the blend from cultivated strawberry included 4-methoxybenzaldehyde as the major component, while the major component in the FV blend mimicking wild strawberry was α-muurolene. The other five components in these two synthetic blends were the same, consistent with the observation that SBW shows no preference for flowering plants of either species.

However, none of the compounds in the two six-component blends increased the preference of SBW when tested as a single compound added to non-flowering plants. This indicates a synergistic action of the volatiles, a common phenomenon in insect behavioral responses to host plant volatiles (Bruce and Pickett 2011; Richards et al. 2016; Sarkar et al. 2017). Olfactory synergism between floral volatiles is less frequently reported compared to odors of vegetative plant parts, possibly due to the activity of individual floral components rarely having been examined (Metcalf et al. 1995; Richards et al. 2016).

4-Methoxybenzaldehyde, the major component of floral volatiles from *F. x ananassa*, actually reduced the attractiveness of non-flowering plants when added at the highest dose of 1000 ng. A similar phenomenon was reported by Webster et al. (2010), showing that individual components of attractive blends of host-plant volatiles can have repellent activity when presented at higher than natural doses and outside the context of the natural host blend.

1,4-Dimethoxybenzene increased the attractiveness to SBW of non-flowering *F. vesca* plants when added as a single compound. This compound was previously identified as a major component of floral volatiles of *F. viridis* (Blažytė-Čereškienė et al. 2017; Wibe et al. 2014) and increased the attractiveness of SBW aggregation pheromone in field trials (Baroffio et al. 2018; Wibe et al. 2014). This provides encouragement that our bioassay results are relevant to the field. We plan to test the active blends from the bioassays for attractiveness to SBW and/or synergism of the aggregation pheromone in field trials.
